# Realizing Promising Educational Practices in Academic Public Health: A Model for the Scholarship of Teaching and Learning

**DOI:** 10.3389/fpubh.2021.750682

**Published:** 2022-01-31

**Authors:** Leah C. Neubauer, Cheryl Merzel, Elizabeth M. Weist, Jaime Antoinette Corvin, Allan Forsman, Jacquie Fraser, Heather L. Henderson, Leslie J. Hinyard, Karin Joann Opacich, Miryha G. Runnerstrom

**Affiliations:** ^1^Feinberg School of Medicine, Northwestern University, Chicago, IL, United States; ^2^School of Global Public Health, New York University, New York, NY, United States; ^3^Association of Schools and Programs of Public Health (ASPPH), Washington, DC, United States; ^4^College of Public Health, University of South Florida, Tampa, FL, United States; ^5^College of Public Health, East Tennessee State University, Johnson City, TN, United States; ^6^Programs in Public Health, Walden University, Minneapolis, MN, United States; ^7^School of Public Health, West Virginia University, Morgantown, WV, United States; ^8^School of Medicine, Saint Louis University, St. Louis, MO, United States; ^9^School of Public Health, University of Illinois at Chicago, Chicago, IL, United States; ^10^Program in Public Health, University of California, Irvine, Irvine, CA, United States

**Keywords:** pedagogy, substantive topics, teaching and learning, academic public health, faculty, SoTL conceptual framework

## Abstract

This paper presents a conceptual framework and critical considerations for the scholarship of teaching and learning (SoTL) in academic public health. Academic education for public health has undergone significant transformation over the last two decades as the demand for responsive and innovative public health pedagogy and training for preparing graduates to deploy an increasing array of skills has grown. The authors suggest that the role of schools, administrators, faculty, and educational staff in developing promising practices for teaching and learning in public health involves an articulated conceptual framework to guide the development and dissemination of scholarly, pedagogical innovations. Building on seminal philosophical foundations of SoTL, the authors conceptualize SoTL from the foundational belief that knowing and learning are communal tasks and that faculty are both scholars and learners in the practice of education. The paper advocates for SoTL as a form of engaged practice and scholarly inquiry that exists in contextually rich, diverse educational environments that abounds with uncertainty. SoTL is guided by an educational philosophy, values, and learning theories that envision educators critically examining themselves, their teaching practice, scholarly literature, and students' learning to improve their teaching, enhance learning, and promote further inquiry. The authors suggest that SoTL involves the search for multiple forms of evidence and fosters dialogues on multiple interpretations and perspectives of the most promising practices of teaching and learning. The authors advocate for the term promising practices as an outcome of SoTL that supports and nurtures ongoing scientific discovery and knowledge generation, instead of supporting the search for best-ness in teaching and learning endeavors. SoTL should occur across formal, informal, and nonformal education

## Introduction

This paper presents a conceptual framework and model and proposes critical considerations for advancing the scholarship of teaching and learning (SoTL) in academic public health. In doing so, the authors assert that advancing education and training in academic public health demands that individual educators, academic institutions, and the field of academic public health examine and name their paradigms, schools of thought, practices, programs, policies, and structures regarding SoTL, how they see themselves in relation to this scholarship, and what methods should be used to discover, apply, and disseminate it. The proposed conceptual framework synthesizes previous SoTL scholarship by integrating multiple dimensions (specific components and key processes) and three outcomes of SoTL into a single model. The foundational Model for Scholarly Teaching in Action and Practice presented in the following pages aims to guide the systematic and comprehensive assessment, development, implementation and evaluation of pedagogical scholarship for spurring innovation and influencing teaching practices at three levels of impact: individual educators, academic institutions (departments, programs, colleges, etc.), and the field of academic public health.

Pedagogical scholarship is more than a discussion of teaching and learning methods and techniques. Transformations in education also invite academic institutions to consider how teaching, learning, and scholarly work focused on pedagogy are conceptualized, implemented, evaluated, and rewarded. Institutionalizing SoTL requires that universities reexamine the role, resources, and supports provided to enhance and scale teaching, learning, and SoTL.

The demand for responsive and innovative education in public health is immense. Higher education is continually challenged to rethink and reinvent education. Academic public health is heeding this challenge and reforming how we educate the next generation of professionals to meet the ever-evolving demands of twenty-first century public health. New directions are reflected in the Framing the Future project convened by the Association of Schools and Programs of Public Health's (ASPPH). The project's report, “Framing the Future: The Second 100 Years of Education for Public Health” (1) calls for the adoption of new and innovative approaches to prepare public health leaders. Subsequent accreditation criteria issued by the Council on Education for Public Health (CEPH) (2) have further prompted accredited schools and programs to embark on fresh and ongoing assessment of their curricula and educational outcomes.

Educators in public health are called to deliver foundational, dynamic, and complex information. Sullivan and Galea (3) suggest that “public health educational programs must prepare graduates to engage with emerging public health issues with knowledge, skills, humility, and personal and professional confidence” and that schools, administrators, faculty, and educational staff play a role in developing optimal practices for teaching and learning in public health. Meeting this demand requires critical understanding of the science of teaching and learning and the ability for learners and teachers to connect on a deeper level, sharing knowledge, and ensuring critical thinking and a more holistic understanding of the skills needed to combat twenty-first century public health problems. Education in public health requires an integrated, systems-thinking approach to address the complex problems facing populations.

In September of 2018, ASPPH set forth to convene a task force of member leaders in the scholarship of teaching and learning to identify assets, gaps, and priorities in this area. Four working groups were formed, one of which was focused on the development of a conceptual model of SoTL for the purpose of guiding academic public health institutions in the support of scholarly work in pedagogy. This entity, entitled the Conceptual Framing working group, convened from 2019 to 2021 with the ultimate aim of improving the quality of teaching and learning in public health. In this paper, the working group discusses guiding pedagogical paradigms and principles and presents a conceptual model of Scholarly Teaching in Action and Practice (STAP) for educators to apply SoTL in academic public health.

## Pedagogical Framework for the Scholarship of Teaching and Learning in Public Health

The realities for the twenty-first century student involve rapidly evolving technology and the immediate availability of and access to knowledge and information. Students also require related, but distinct, skills to synthesize and apply that information. The evolution of the “classroom” includes the galvanization of research and education, ensuring that public health content and curricula are founded on evidence and instruction is based on the neuroscience of how learners learn and the sociocultural perspectives of learning, recognizing the interdependence of individual and social processes of learning and knowledge-creation (4).

As cognitive researchers influence education, the science of learning has placed primary emphasis on “learning with understanding” (5). A hallmark of SoTL is active learning, which requires students to engage with knowledge and to apply that knowledge, ultimately fostering deep understanding and that allows students to transfer knowledge to other contexts. Based upon the underlying tenets of experiential learning, SoTL encourages teachers to engage students and allow for the application of knowledge and conceptual understanding to real-world problems, case studies, or other scenarios.

There are four basic principles underlying SoTL. SoTL: (a) treats teaching “as a form of inquiry into student learning” (6), with concern for students and how students learn as pivotal to the process; (b) is based upon “deliberate design” (6, 7), which focuses on the most effective design for learning and encourages engagement and interaction to achieve learning; (c) requires systematic implementation and evaluation to ensure optimal student learning and the advancement of teaching; (d) must be accessible to scholars within the field, for encouraging scholarly consumption of and eventual commitment to the practice of more evidence-based modes of teaching and learning as well as the production of scholarly dissemination of experience and findings.

SoTL is thus guided by an educational philosophy, values, and learning theories where educators critically examine themselves, their teaching practice, scholarly literature, and students' learning to improve their teaching, enhance learning, and promote further inquiry. The authors suggest that SoTL endeavors involve the search for multiple forms of evidence and foster dialogues and deliberations on multiple interpretations and perspectives of the most promising practices of teaching and learning. SoTL both invites the need to evaluate pedagogical practices and disseminate those evaluations and key learnings in the larger body of literature.

The authors advocate for the term *promising practices* (8, 9) as an outcome of SoTL that supports and nurtures ongoing scientific discovery and knowledge generation, instead of supporting the search for best-ness (e.g., best practices) in teaching and learning endeavors. Building on the work of Boyer (10), Shulman and Shulman (11), Brookfield (12) and Patton (9), the authors conceptualize SoTL from the foundational belief that (a) knowing *and* learning are communal, contextually-responsive tasks, (b) faculty are both scholars and learners in the practice of education, and (c) “best practices aren't” (9). The authors suggest that the path to developing *promising practices* for teaching and learning in public health involves an articulated conceptual framework to guide the development and dissemination of scholarly, pedagogical innovations. *Promising practices* presume that teaching demands context-relevant adoption and is rooted in humility and uncertainty; a movement away from the *certainty* and *universality* conveyed in “best practices” (9, 13). Faculty committed to SoTL must closely evaluate pedagogical practice and apply approaches to researching, evaluating, and enhancing student learning that attend to the context of the real-world practice of teaching (14).

## A Model for Scholarly Teaching in Action and Practice

As educators, we aim for dual, interrelated goals—improving the quality of our teaching and enhancing student learning outcomes. Scholarly teaching provides a framework for attaining both objectives. Trigwell et al. (15) note that the aim of scholarly teaching is “to make transparent how we have made learning possible” (p. 156). Achieving this straightforward yet complex aim requires a process of: (1) applying pedagogical theory and evidence-based methods to our teaching; (2) examining the effectiveness of this application in our teaching practice; and (3) reflecting on the meaning of the findings for future teaching and disseminating the key learnings (15). Many educators informally apply this approach and thus, in practice, are engaged in some degree of evidence-based teaching.

The Model for Scholarly Teaching in Action and Practice (STAP) aims to assist educators in pursuing and implementing a systematic process of teaching. The model is based on the premise that SoTL reflects a continuum, ranging from an orderly examination of our own teaching practices to rigorous study of pedagogical theory and methods. Our aim is to encourage the practice of SoTL at multiple levels, including individual educators, institutions, and the field of academic public health by making transparent the processes for implementing scholarly teaching and identifying meaningful outcomes for assessing pedagogical research and practice.

**Figure 1:**
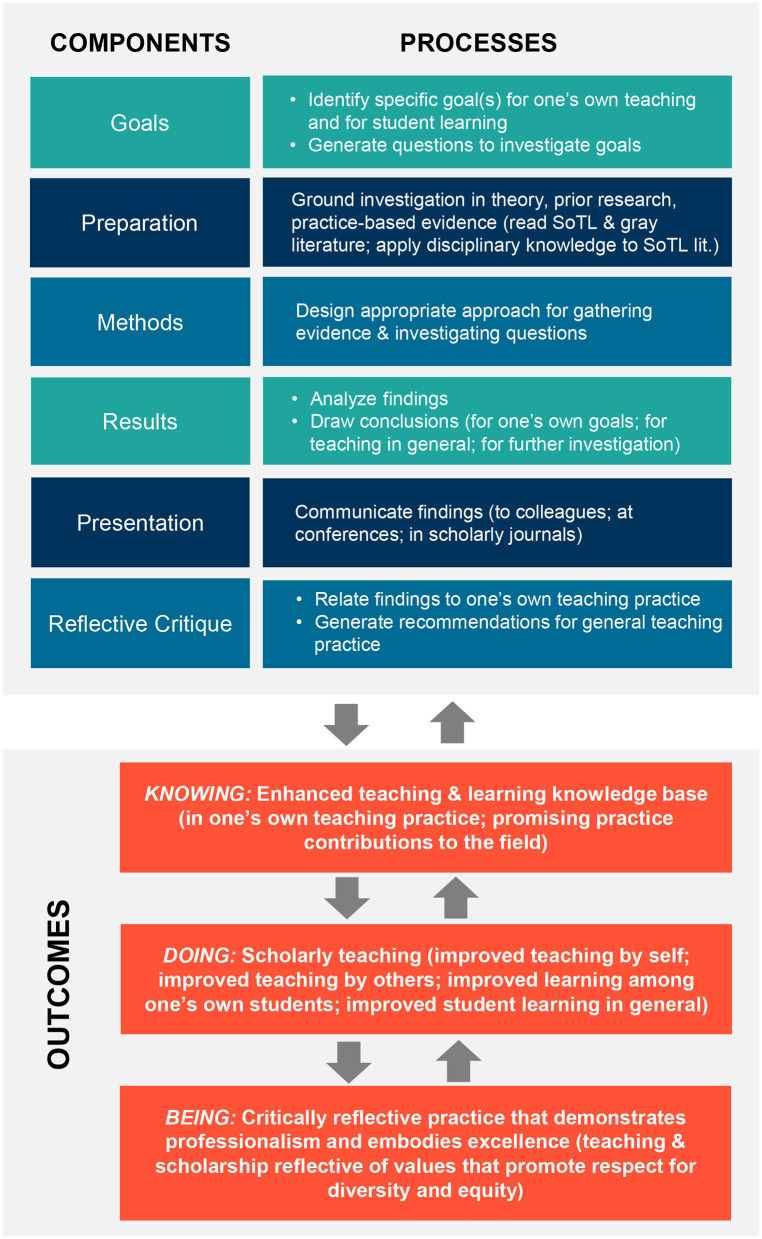
Model for scholarly teaching in action and practice (STAP) [adapted from Bishop-Clark and Dietz-Uhler (16), Cox (17), Glassick et al. (18), and Trigwell et al. (15)].

The STAP model (Figure 1) delineates: (1) the main components for engaging in SoTL; (2) corresponding processes; and (3) resulting outcomes related to scholarly teaching practice. The model is a synthesis of approaches and frameworks for SoTL found in the general pedagogical literature (15–18) and outcomes reflected in the educational goals of public health (19). Essential components, key processes, and multidimensional outcomes from this literature are integrated in a unified action-oriented model that can be used to guide implementation of scholarly teaching. We discuss each aspect of the model in more detail below.

### Components of Scholarly Teaching

The six components of the model provide a framework characterizing the main elements of scholarship as delineated by the frequently cited work of Glassick et al. (18) for the Carnegie Foundation for the Advancement of Teaching. The components identify the essential criteria that educators must address when conducting a SoTL investigation, including:

1) Goals: This investigation should have clearly defined, realistic, and achievable goals and objectives.
2) Preparation: The educator adequately prepares for the investigation by basing it on existing scholarship, evidence-based practice, and other sources of knowledge
3) Methods: The educator selects appropriate methods to investigate achieving the goals and objectives.
4) Results: Findings should indicate whether goals and objectives were achieved, and contribute to the field.
5) Presentation: The work is communicated in a format and style suitable to intended audiences, with appropriate documentation.
6) Reflective critique: Educators critically evaluate their own work and use the evaluation to improve future work.

### Processes for Scholarly Teaching

Each component of the scholarly teaching model is operationalized through a series of processes that broadly reflect the elements and steps of a research project (16). The processes encompass investigating questions relevant to one's own teaching and may also extend to contributing to pedagogical knowledge and practice in one's field (15). Reflection is essential as a means of deepening one's understanding of how to apply knowledge gained through the investigation (15). The scholarly teaching processes, which correspond to each component in the model, include:

1) Goals: Identify specific educational goals that the educator is interested in attaining and, toward this end, generating questions to investigate to help reach the goals. Goals and questions may be limited to one's own teaching needs or could encompass future dissemination to the field.
2) Preparation: Ground the investigation in existing scholarship and practice-based evidence relevant to the aims. This involves a systematic examination of the SoTL literature and review of cases from actual teaching practice (e.g., syllabi, assignments, etc.) viewed through the lens of the academic discipline(s) in which the teaching occurs.
3) Methods: Develop a sound design for the investigation that allows for gathering information to answer the questions identified. This approach could encompass varying types of evidence and methods (e.g., randomization, quasi-experimental designs, mixed methods, case studies, etc.);
4) Results: Analyze findings to answer the questions posited for the investigation.
5) Presentation: Disseminate results to various audiences (e.g., colleagues at one's home institution, conferences, community partners, and/or peer-reviewed journals).
6) Reflective critique: Reflect critically on the findings to identify implications for one's own teaching practice and to develop generalized recommendations for the field.

### Outcomes for Scholarly Teaching

As noted above, the aim of scholarly teaching is to advance the quality of teaching, leading to better student learning. These objectives help define meaningful outcomes to measure as part of a SoTL investigation. The model classifies outcomes to reflect three dimensions of pedagogical sequelae, drawing on principles shaping education for public health: knowing, doing, and being (19). *Knowing* addresses the aim of this scholarship, namely to add to the knowledge base for teaching and learning, and to contribute promising practice to scholarly teaching practice (17). *Doing* scholarly teaching works in all settings and can be deployed and assessed by examining improvements in one's own teaching, increased learning among one's own students, and improved student learning in general, and is shared via dissemination to the field (15). *Being* involves engaging in critical reflection to ensure that teaching and scholarship represent important public health values, including respect for diverse individuals and communities and promoting equity in our professional practice. These outcomes are part of an iterative feedback process that contributes to the ongoing investigation, practice, and improvement of scholarly teaching and which contributes to professionalism and excellence.

## Practical Implications: Applying the STAP Model

There are myriad ways to use the STAP model to engage in scholarly teaching in academic public health. From an assessment of how group work in a course impacts student learning to an evaluation of program-wide learning outcomes, the STAP model is a framework to guide pedagogical inquiry at multiple levels.

Guidelines and an example for application of the STAP model are provided in Table 1. The table includes guidance for implementing each of the model's six processes. The example shows how the STAP model could be used to assess changes in students' sense of belonging in a public health major through participation in a yearlong professional development course for first-generation college students. Several possible mechanisms for disseminating the results of the example research project and how the results could be used to inform programmatic changes to support first generation college students' sense of belonging are detailed.

**Table 1:** Case example of putting the STAP model into practice.

**Components**	**Processes**	**Example**
Goals	Generate questions to address regarding your courses, teaching methods, and/or educational program.	Does a yearlong professional development and leadership course for first-generation college students improve their sense of belonging in the undergraduate public health major?
Preparation	Review resources and sources of information (e.g., peer-reviewed literature, disciplinary knowledge, and teaching practices prior experiences) to help you answer your questions.	Review the literature on college students' sense of belonging and on challenges faced by first-generation college students to understand how these factors impact student success, persistence, and engagement.
Methods	Select appropriate research methods (e.g., student surveys, analysis of student learning outcomes, mixed methods studies) to help you answer your questions.	At the start and the conclusion of the course, survey enrolled students on their sense of belonging in the major.
		Conduct semi-structured key informant interviews of first-generation students who recently left the public health major to understand why they chose to leave and if/how sense of belonging may have contributed to their decision.
Results	Use analytical approaches and tools to appraise and synthesize your findings and for making recommendations.	Analyze quantitative survey data using statistical methods.
		Conduct inductive analyses of qualitative interview transcripts to identify themes that help answer the research question.
Presentation	Identify target audiences and formats for presenting your conclusions (e.g., publications, conferences, faculty meetings).	Share findings with faculty, staff, and administrators in one's school or program.
		Share findings with university teaching and learning research unit, if applicable.
		Present findings at pedagogy- and/or public health-focused conferences.
		Submit a manuscript of findings to a pedagogy- and/or public health-focused journal.
Reflective critique.	Place findings in the context of your teaching practice or your educational program's goals and generate recommendations for teaching or programmatic practice	Use study results to recommend programmatic changes to better support first-generation students' sense of belonging in the public health major.

## Challenges and Considerations

Designing and implementing SoTL brings challenges. Addressing the evolving needs of students and the public health workforce demands ongoing, responsive, and nimble attention. Building scholarship in teaching and learning requires appreciation of the determinants of learning and the context in which teaching occurs. The SoTL framework can assist with addressing these challenges on the level of individual educators, academic institutions, and the field of academic public health. Implications for each level are detailed below.

*For individual educators*, finding the time, motivation, and institutional support for SoTL can be challenging. Educators need opportunities to identify innovative approaches and promising practices that will assist them to fine tune skills and disseminate knowledge. Some approaches to support individual educators include incentivizing uptake of continuing education opportunities, engaging with peer networks, participating in conferences, and collaborating with teaching professional development centers.

*For institutions*, it is vital for departments, programs, schools, and entire universities to examine how policies, procedures, and institutional culture support or discourage SoTL. This includes consideration during promotion and tenure processes. To foster SoTL, institutions should include and must examine pedagogical scholarship in promotion and tenure considerations. Doing so would require viewing SoTL efforts as scholarship on an *equal footing* with other forms of research and would underscore the importance of teaching and learning in scholarship. Considerations must also be made in terms of resource capacity. Most faculty are not trained in pedagogy. Institutions need to offer expert support to faculty to engage in pedagogical scholarship. To support these efforts, institutions should also consider offering research funding and faculty development opportunities related to SoTL.

*For the field of academic public health*, improved educational practices often result from changes to accreditation standards. The Council for Education in Public Health (CEPH), co-sponsored by ASPPH and the American Public Health Association, is an important partner that presents a potential lever of change in support of SoTL. One of CEPH's recent revisions in its *Accreditation Criteria for Schools of Public Health and Public Health Programs* specifically “E4 Faculty Scholarship,” adds “advancing the scholarship of teaching and learning” as a viable option for faculty research (20). This addition represents an example of a facilitating factor that practitioners of SoTL may draw upon in substantiating their contributions as valuable to their host institution and to the field of academic public health.

## Conclusion

Education in public health has transformed greatly over the past decade. Opportunities to develop a SoTL agenda for individual educators, academic institutions, and the field of academic public health are timely and relevant. The SoTL perspective envisions educators in multiple roles in the art and science of teaching and learning: *scholars, learners, practitioners, and advocates*. Educators in public health may engage with SoTL in multiple ways across different levels, from teacher-focused and informal, to student-focused and reflective (21). SoTL in the field of public health is not a one-size-fits-all endeavor. Our emphasis on *promising practices* both reflects current priorities in academic public health and expands the arena for critically engaging in scholarly pedagogy. Wide adoption of the model is posited to support increased individual, institutional, and disciplinary opportunities to enhance learning outcomes and advance the scholarship of teaching and learning. Approaching public health pedagogy through a SoTL lens has multiple benefits for both students and faculty including enhanced student learning outcomes, and engaged, reflective instruction.

The STAP model encourages academic public health as a field to prioritize and develop its own tradition of scholarly inquiry. Schools and programs of public health stand to benefit from active engagement in pedagogical research and the dissemination and implementation of educational research findings (22). University leaders and academic administrators must consider the types of organizational commitments and culture shifts needed to facilitate meaningful adoption of responsive teaching and learning and SoTL in ever-changing educational environments (23). A deepened SoTL community and appreciation for all forms of scholarly pedagogical inquiry will further advance education and training in public health and, by extension, enhance the profession. Among a slew of responsibilities, faculty are called to re-examine the paradigm of education for public health and develop relevant, inclusive, culturally competent curricula (24). The STAP model provides a roadmap for developing the habits, processes, and systems needed to advance a SoTL for academic public health.

## Data Availability Statement

The original contributions presented in the study are included in the article/supplementary material, further inquiries can be directed to the corresponding author/s.

## Author Contributions

LN, CM, and MR conceived the conceptual framework for the paper. CM led development of the conceptual model and was supported by LN, MR, and EW. LN led all phases of the manuscript development. EW, JC, AF, JF, HH, LH, and KO provided critical feedback to the conceptual model and contributed to all phases of the manuscript development. MR conceived the idea and co-developed the conceptual model. All authors contributed to the article and approved the submitted version.

## Conflict of Interest

The authors declare that the research was conducted in the absence of any commercial or financial relationships that could be construed as a potential conflict of interest.

## Publisher's Note

All claims expressed in this article are solely those of the authors and do not necessarily represent those of their affiliated organizations, or those of the publisher, the editors and the reviewers. Any product that may be evaluated in this article, or claim that may be made by its manufacturer, is not guaranteed or endorsed by the publisher.
